# Rapid and reproducible generation of glioblastoma spheroids for high-throughput drug screening

**DOI:** 10.3389/fbioe.2024.1471012

**Published:** 2024-12-18

**Authors:** Christian Bach, Annegret Glasow, Rainer Baran-Schmidt, Henry Oppermann, Christoph Bach, Jürgen Meixensberger, Erdem Güresir, Frank Gaunitz

**Affiliations:** ^1^ Department of Neurosurgery, University Hospital Leipzig, Leipzig, Germany; ^2^ Department of Radiotherapy and Radiooncology, University of Leipzig, Leipzig, Germany; ^3^ Institute of Human Genetics, University of Leipzig, Leipzig, Germany

**Keywords:** glioblastoma, spheroids, viability assay, high-throughput drug screening, ultra-low-attachment plate

## Abstract

Identifying new substances that could potentially be used for tumor therapy and the precise analysis of their spectrum of action requires models that are as similar as possible to the tumor present in the patient. Traditionally, two-dimensional (2D) cell cultures are used. However, these only resemble solid tumors to a limited extent. More realistic *in vivo* models, such as tissue cultures, which are invaluable for the final analysis of the effect of new substances, are unsuitable for high-throughput screening (HTS), such as substance library screening. Therefore, we addressed which parameters need to be optimized to produce 3D cultures suitable for HTS using established tumor cell lines and ultra-low attachment plates, and we tested which experimental parameters need to be considered. In our studies, we have focused on cell lines from gliomas. Gliomas are incurable tumors of the central nervous system and are the subject of intensive research. Our studies used ten glioma cell lines from which we generated spheroids using ultra-low attachment plates. We then determined the spheroid size as a function of the initial cell number and the culture time. We analyzed cell viability using propidium iodide staining, evaluated the effects of temozolomide and radiation on spheroids, and compared the effect to that on 2D cultures. We found that spheroid size correlated linearly with the initial cell number. Fewer cells (250–500) generally resulted in better growth than a higher number. However, not all cell lines produced growing spheroids at all. The spheroids had an outer layer of living cells and an inner core of dead cells. The size of the inner core of dead cells was different in the various cell lines and developed differently during the incubation period. Radiation affected spheroids more than 2D cultures, especially at higher cell densities. Our results provide insight into using glioma cell lines to form spheroids as model systems. We have identified initial cell numbers as a critical parameter for their effective use in research, offering a hopeful outlook for tumor therapy research and drug development.

## 1 Introduction

For decades, research laboratories have been cultivating cells in suspension or, more frequently, adherently in flasks or culture dishes to carry out various studies. In many cases, 2-dimensional (2D) cultures from adherently growing primary cultured cells from tumor tissue or cell lines are used to identify substances potentially active as tumor therapeutics. While this model, coupled with modern high-throughput screening (HTS) techniques, allows for large-scale testing of substances, it has significant limitations. Many effective substances in 2D cultures rarely enter the clinic, failing in downstream experiments with more sophisticated models, e.g., animal models, or ultimately in clinical trials (Sun et al., 2022). This observation underscores the need for experimental models to better predict the usability of substances for therapeutic purposes, especially for treating different types of cancer. Among the most important aspects is that 2D cultures do not adequately reflect the gradients of oxygen, nutrient components, or metabolites in a solid tumor. In addition, cell-cell and cell-matrix interactions are missing (Jensen and Teng, 2020). Various methods, such as the production of organoids or organotypic slice cultures, have been developed to address these aspects (Foglizzo et al., 2022). Despite the apparent advantages of these models compared to two-dimensional cultivation, the production of these models is very time-consuming and labor-intensive. As a result, only a relatively small number of individual tests can be carried out compared to 2D culture, which makes it difficult or even impossible to use HTS or even to test many different concentrations of a substance in individual tests or combinations with other substances. In addition, there is the problem of having methods to measure the efficacy of the tested substances with acceptable z-factors, such as cell-based assays in 2D cultures (Bar and Zweifach, 2020). Another issue is that although using patient tissue for organoids or organotypic slice cultures offers a more patient-oriented situation, the impossibility of reproducibility significantly limits the possibility of making a general statement about a substance’s therapeutic potential.

Glioblastoma (IDH-wildtype) is one of the most challenging tumors for developing new therapeutics. This tumor of the central nervous system has a median survival time of 16.9 months after diagnosis and application of the current standard therapy (Brown et al., 2022). Therefore, this tumor entity represents a particular challenge. Although experimental models for glioblastoma, such as organotypic slice cultures, have been developed (Merz et al., 2013), the problem remains that these models are not suitable for performing reproducible HTS. For this reason, we wondered whether spheroid cultures of glioblastoma cell lines, which can be produced relatively quickly and in large numbers using ultra-low attachment (ULA) plates, could bridge the gap between simple 2D cultures and higher models, such as organotypic slice cultures. To this end, we analyzed cells from 10 different glioma cell lines for their ability to form spheroids. We investigated under which conditions they do this optimally and how the growth behavior and properties of the spheroids present themselves concerning the proportion of living and dead cells. Finally, spheroids of selected cell lines were subjected to a “standard therapy” as proof-of-principle, and the viability of the spheroids was compared with the viability of equivalently treated cells in 2D culture using a quantitative assay.

## 2 Materials and methods

### 2.1 Reagents

Unless otherwise stated, all chemicals were purchased from Sigma Aldrich (Taufkirchen, Germany).

### 2.2 Cell culture

The cell lines 1321N1 and U-251MG were obtained from Sigma Aldrich (Taufkirchen, Germany), and the cell lines U87, T98G, and LN229 from the American Type Culture Collection (ATCC; Manassas, United States). MZ54, G55T2, and MZ18 were initially obtained from Donat Kögel (Frankfurt, Germany), and the lines LN405 and U-343MG from the German Collection of Microorganisms and Cell Cultures (DMSZ; Braunschweig, Germany). Table 1 provides a detailed description of all cell lines used. Cells were cultured at 37°C, 5% CO_2_, and 95% air in DMEM (Dulbecco’s Modified Eagle Medium containing 4.5 g glucose/mL) supplemented with 2 mM Glutamax, 1% Penicillin/Streptomycin (all from Gibco Life Technologies, now Thermo Fisher Scientific, Darmstadt, Germany) and 10% Fetal Bovine Serum (FBS; Biochrom GmbH, Berlin, Germany), hereafter referred to as “culture medium.” This culture medium was used for all experiments, including the formation of spheroids. Confluence was checked by phase contrast microscopy.

**TABLE 1:** Cell lines employed for the construction of spheroids.

Name	RRID	Sex	Age	Diagnosis	Grade	% Ident	IDH
T98G	CVCL_0556	Male	61	Glioblastoma	4	100.00	WT
LN405	CVCL_1378	Female	61	Glioblastoma	4	100.00	WT
U87	CVCL_0022	Male	NA	Glioblastoma	4	98.46	WT
LN229	CVCL_0393	Female	60	Glioblastoma	4	95.60	WT
U-343MG	CVCL_4773	Male	54	Glioblastoma	4	98.30	WT
MZ18	CVCL_M401	Male	72	Glioblastoma	4	NA	WT
MZ54	CVCL_M406	NN	NA	Glioblastoma	4	NA	WT
1321N1	CVCL_0110	Male	47	Astrocytoma	NA	98.11	WT
U-251MG	CVCL_0021	Male	75	Astrocytoma	NA	100.00	WT
G55T2	CVCL_BW88	Male	65	Anaplastic Astrocytoma	4	NA	WT

The table lists the cell lines used in the study, and the corresponding *Cellosaurus* accession numbers are indicated (Bairoch, 2018). When information about STRs, was available, cell lines were genotyped (Genolytic GmbH, Leipzig, Germany and DSZM, Braunschweig, Germany), and the degree of identity is indicated as “% Ident.” All cell lines were IDH, wild type as determined by sequencing (Paul-Flechsig-Institute of Pathology, Leipzig, Germany). NA: No data available. Note: G55T2 was adapted to *in vivo* growth by subcutaneous passages of a grade 4 GBM, line designated NCE-G55 (Westphal et al., 1994) in nude mice (Eckerich et al., 2009).

### 2.3 Spheroid formation and 2D culture

Single cells were harvested in their exponential growth phase at 75%–85% confluence using Accutase (Gibco), and the cell number was determined using an automatic cell counter (TC10™ automatic cell counter; Bio-Rad Laboratories GmbH, Feldkirchen, Germany). Spheroids were developed in 96-well ultra-low attachment plates (Corning^®^ 96-well Black/Clear Round Bottom Ultra-Low Attachment Spheroid Microplate, with Lid, Sterile, Corning Incorporated, Corning, NY, United States). A specific number of cells (see details for each experiment) were pipetted into each well in 150 µL of culture medium. The plates were centrifuged at 241 × g for 5 min, transferred to a cell incubator, and incubated at 37°C, 5% CO_2_ and 95% air. (Note: The seeding of cells is day zero). Every 3–4 days, 50% of the medium was removed, and fresh medium was added. 2D cultures were performed in 96-well plates (µClear; Greiner BioONE, Frickenhausen, Germany) under the same culture conditions.

### 2.4 Staining and imaging of spheroids and cells

Staining with propidium iodide (PI) was performed by adding 0.1 μg/mL PI to the cells at the beginning of the experiments. We used a concentration well below the commonly used concentration of 0.5 μg/mL (or even 10 μg/mL) to avoid toxicity by PI (Krämer et al., 2016), continuously monitoring necrosis without producing artifacts by staining. Imaging was performed with a BZ-X810 Microscope (Keyence, Neu-Isenburg, Germany), using a TexasRed-Filter (OP-87765, Keyence, EX 560/40 nm, DM 585 nm, BA 630/75 nm) for PI fluorescence staining. Phase contrast and PI images were taken at ×20 magnification.

### 2.5 Determination of spheroid diameters and volumes

The radii of the spheroids were measured using the BZ-X800 Analyzer Software (Keyence). Spheroids’ total volume and the inner core of dead cells (stained by propidium iodide) within the spheroids were calculated based on the radii (r) according to the following formula:

$$V=\frac{4}{3}\pi r^{3}$$

The BZ-X800 Analyzers Stitching Tool generated a single image for measuring spheroids larger than a field of view. (Note: In rare cases, when spheroids deviated significantly from a spherical appearance, these structures were omitted from analysis.) At this point, it should be noted that our oversimplified method of determining spheroids’ volume might need to be revised to determine the response of spheroid volume to drugs. However, our method proved sufficient to show the relation between volume and cell number and volume and incubation time.

### 2.6 Determination of the volume of individual cells

The size of individual cells from different cultures was determined from cells harvested in their exponential growth phase at 75%–85% confluence. Trypan blue staining was employed to exclude dead cells from the analysis. The cells were applied to Dual-Chamber Cell Counting Slides (Bio-Rad) to perform microscopy using a Keyence microscope. Cell radii were measured by the BZ-H4XI/Image Cytometer Module (Keyence), which allows setting a lower bound for the exclusion of debris and an upper bound for the exclusion of artifacts. In addition, it allows the separation of doublets. Size determinations were also controlled visually for possible doublets and other artifacts.

### 2.7 Irradiation and temozolomide treatment

From each well containing a spheroid in 150 µL culture medium, 50 µL of the medium was removed and replaced with 50 µL of fresh medium containing 600 µM Temozolomide (TMZ) in DSMO, resulting in 200 µM TMZ per Well. Note: We used a concentration of 200 µM because we evaluated this concentration as appropriate in previous experiments with 2D cultures (Oppermann et al., 2019). However, one should consider that this concentration is ∼3–4 times higher than the peak plasma concentration obtained in a patient receiving the standard dose of 150–200 mg/m^2^/day (Ortiz et al., 2021). As a control, 50 µL of medium containing only DMSO was used. Single dose irradiation (8 Gy) was performed 2 h after TMZ was added to the cells using a 200 kV X-ray machine (Xstrahl, Ratingen, Germany) at a dose rate of 1.43 Gy/min (150 kV, 10 mA).

### 2.8 Determination of cell viability

The viability of cells within a spheroid was visualized using PI staining. The CellTiter-Glo 3D Cell Viability Assay (Promega, Mannheim, Germany) was used according to the manufacturer’s recommendations for quantitively determining spheroids' viability. In brief, an equal volume of CTG-3D reagent was added to each well and then mixed by pipetting and shaking for 5 min. After incubation at room temperature for 25 min, luminescence was determined using a SpectraMax M5 (Molecular Devices, Biberach, Germany). Cell viability in 2D culture was determined using the CellTiter-Glo Cell Viability Assay (Promega) according to the manufacturer’s recommendations.

### 2.9 Statistical analysis and graphical representation

The statistical analysis was performed with SPSS [IBM, Armonk, United States; version: 28.0.0.0 (190)]. For multiple comparisons, a one-way ANOVA with a Games-Howell or Bonferroni *post hoc* test was performed after the test for equality of variance (Levene test). The results were considered statistically significant at a value of *p* < 0.05. Graphical representations were created using OriginPro (2021b; OriginLab Corporation, Northampton, MA, United States) and CorelDraw Graphics Suite 2020 (Corel Corporation, Ottawa, Canada).

The following strategy was used to analyze whether treatment had a statistically different effect on cells in 2D culture than on cells in 3D culture: Cells or spheroids were treated with TMZ, radiation, or a combination of both treatment modalities (all n = 6). The effect of the treatment was compared with untreated control cells or spheroids (defined as 100% viability). After determining the mean value for each treatment group, each value (n = 6 for each modality) was normalized to this mean value. These normalized data were used for a one-factorial ANOVA after a Kolmogorov-Smirnov test, followed by a Levene test to decide whether a Games-Howell or Bonferroni *post hoc* correction should be applied to determine significance.

### 2.10 Artificial intelligence

The correct spelling and grammar were checked using DeepL (DeepL SE, Cologne, Germany) and Grammarly (Grammarly Inc., San Francisco, United States).

## 3 Results

### 3.1 Spheroid size and growth behavior

**FIGURE 1:**
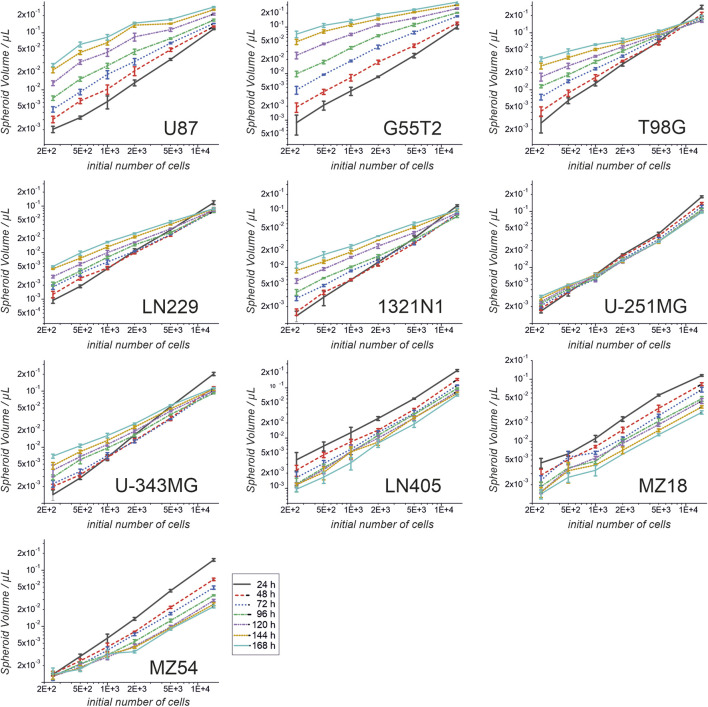
Spheroid volume as a function of the initial cell number and at different cultivation times. Cells from ten cell lines were used to form spheroids. Volumes were determined after 24, 48, 72, 96, 120, 144 and 168 h. Both axes are presented in a log10 scale. Mean and standard deviation were calculated for 4 spheroids (technical replicates; one spheroid in one well; n = 4; T98G; n = 5) at each time point and for each cell number used.

**FIGURE 2:**
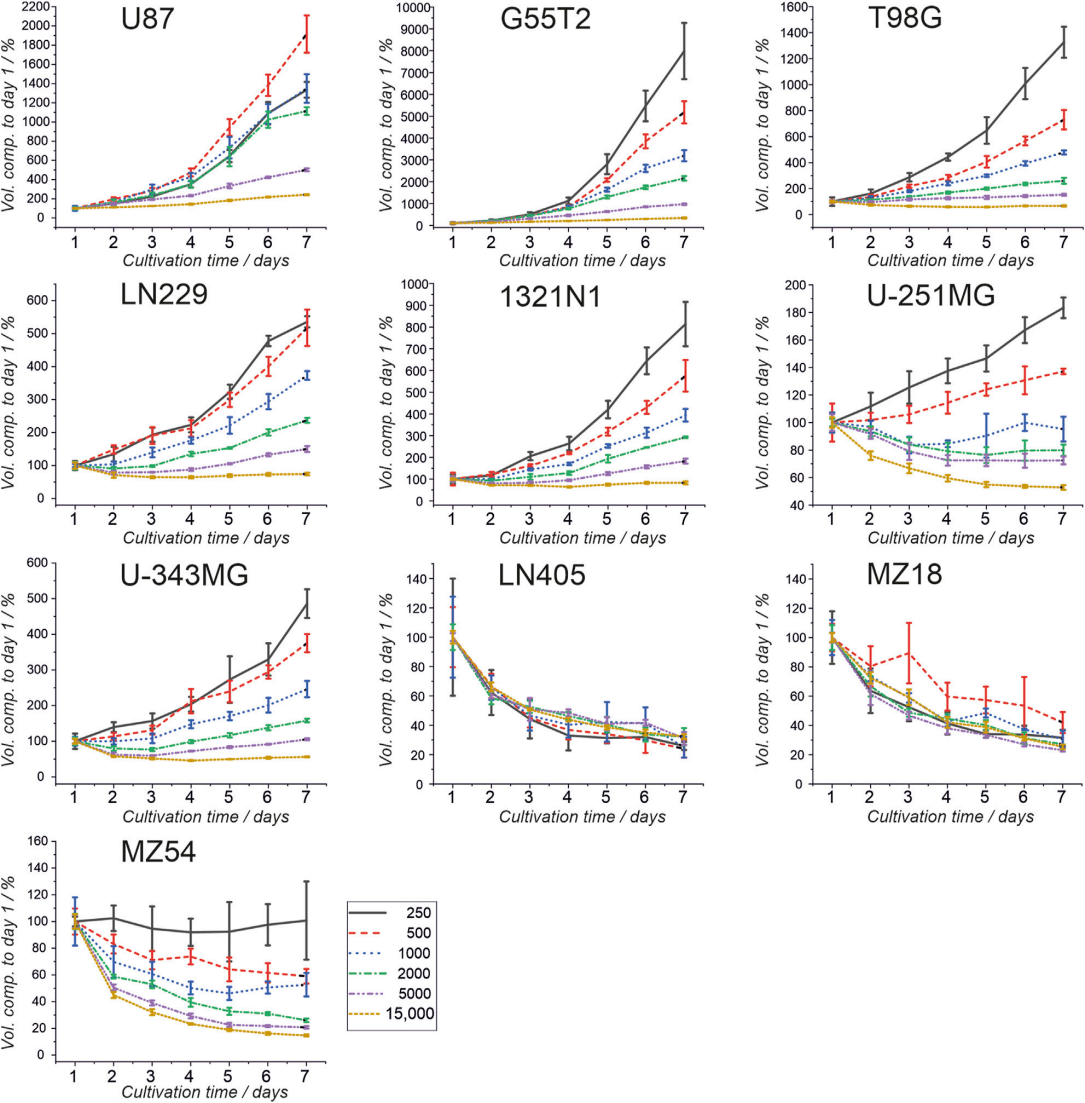
Spheroid volumes as a function of growth time. Cells from ten cell lines were used to form spheroids, and the volumes were determined after 24, 48, 72, 96, 120, 144, and 168 h. Volume after 24 h (day 1) was set as 100%, and all other volumes were compared to the day 1 volume. Mean and standard deviation were calculated for 4 spheroids (technical replicates; one spheroid in one well; n = 4; T98G; n = 5) at each time point and for each cell number used.

First, we investigated the spheroid size as a function of the initial number of cells. Therefore, 250, 500, 1,000, 2000, 5,000, and 15,000 cells were used to form spheroids. The size was measured after 24, 48, 72, 96, 120, 144, and 168 h. The results of the experiment are shown in Figures 1, 2. In addition, pictures from spheroids can be seen in Supplementary Material 1. As can be seen in Figure 1, the size of the spheroids appears to be a linear function of the initial number of cells in this double logarithmic presentation. A linear fit using the original non-logarithmic data confirmed a linear relation in most cases (see Table 2; all adjusted *R* ^2^ values for the linear fit, including intercept and slope are presented in Supplementary Table 1). However, some lines, such as U87, G55T2 and T98G deviate from a linear relation with increasing incubation time. These lines also respond to increased incubation time with an increased slope (pL Spheroid/cell number) of the linear regression curves whereas the other lines show a tendency towards decreased slopes. More important, as shown in Figure 2, which plots spheroid volume as a function of cultivation time, the spheroids of lines LN405, MZ18, and MZ54 do not grow, regardless of the initial number of cells used. The data in Figure 2 also show that growth is generally better with a lower initial number of cells. While spheroid growth of U-251MG cells can only be observed from 250 or 500 cells, spheroids of U87 cells still show some growth, even from 15,000 cells. Although spheroid growth differs between the lines, the best growth can generally be observed at an initial cell number between 250 and 500.

**TABLE 2:** Linear regression fitting of data presented in Figure 1.

	U87	U-251MG	G55T2	U-343MG	T98G	LN405	LN229	MZ18	1321N1	MZ54
24 h	0.99796	0.9855	0.99049	0.99457	0.99191	0.99546	0.99417	0.97498	0.99417	0.99687
48 h	0.99581	0.99458	0.9997	0.99675	0.99882	0.99135	0.99702	0.99464	0.99763	0.99926
72 h	0.98255	0.99318	0.98194	0.99847	0.98684	0.996	0.99919	0.99819	0.997	0.99996
96 h	0.97115	0.99381	0.93321	0.99372	0.95966	0.99907	0.99711	0.9885	0.99769	0.99986
120 h	0.91975	0.99671	0.87858	0.98862	0.94449	0.9979	0.99562	0.99285	0.98756	0.99956
144 h	0.82983	0.99703	0.84264	0.98772	0.96946	0.99911	0.97963	0.99383	0.97502	0.99903
168 h	0.83751	0.9975	0.85283	0.9771	0.96286	0.99445	0.95793	0.9906	0.94242	0.99587

The data presented in Figure 1 was used to perform a linear regression by fitting the non-logarithmic data to the formula y = a + b*x with y: volume of spheroid; a: intercept; b: slope and x: number of cells. Numbers highlighted in green mark *R* ^2^ value >0.98, those highlighted in yellow, *R* ^2^ values between 0.98 and 0.95, and numbers highlighted in red those below 0.95.

### 3.2 Analysis of single-cell volumes and size distribution

**FIGURE 3:**
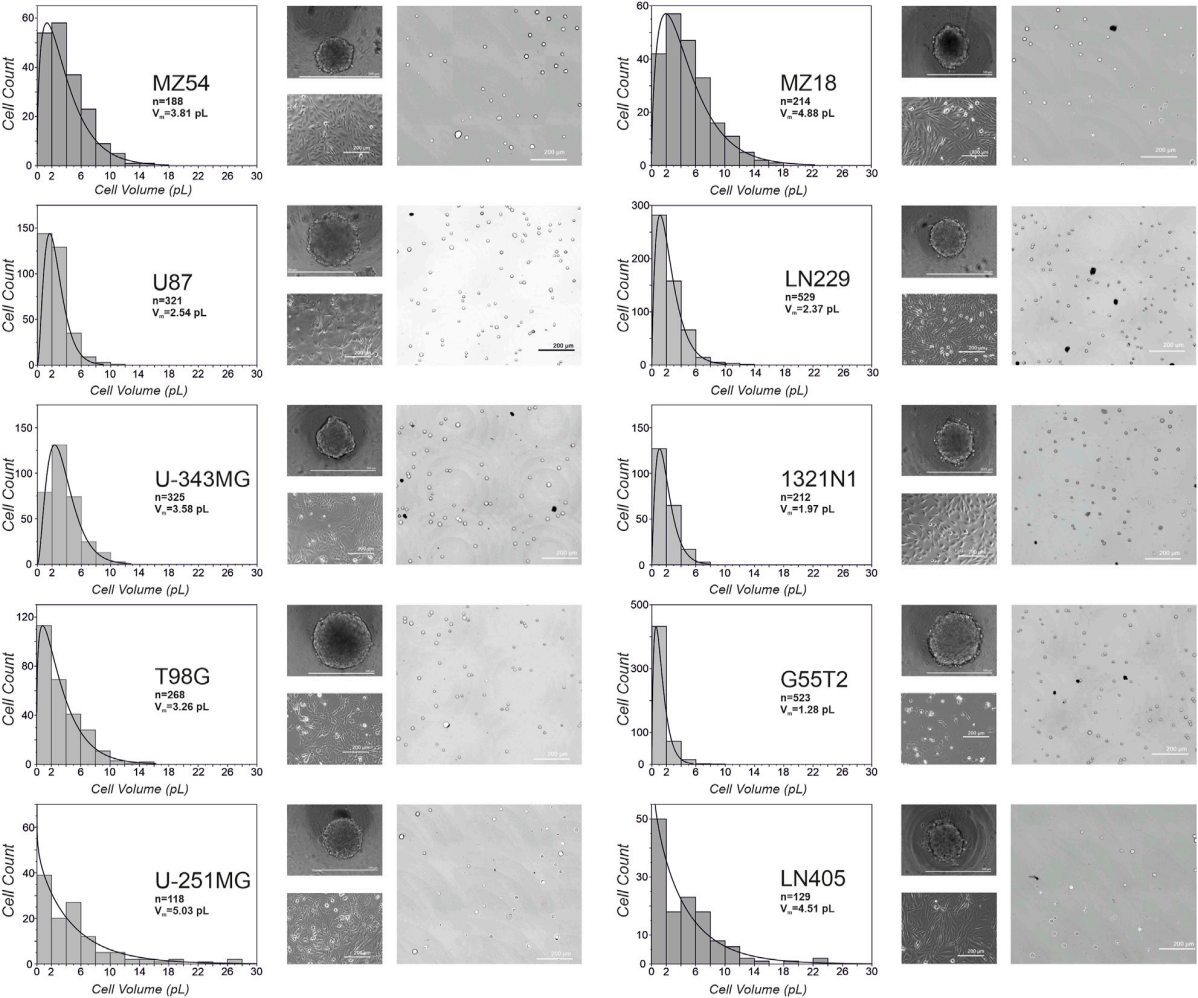
Volumes and size-distribution of cells from different glioma cell lines. Depicted is the distribution of cell volumes for the ten different cell lines used in the study. In addition, microscopic images from spheroids at day 3, phase contrast images of adherent cells and representative pictures from detached cells used for the calculation of volumes are shown. V_m_: average volume of a cell; n: total number of cells analyzed.

As microscopy of cells in 2D culture indicated that cells of different origins differ in volume and size distribution, we wondered whether the volume of the single cells may influence the formation and growth of spheroids. We especially considered, that a cell line that shows a high variety of cell volumes within a culture may form spheroids that have a different growth behavior compared to a cell line with cells that do not show a high variety of different cell volumes. Therefore, we measured cell volume and size distribution of cells after their detachment from culture flasks before they were used to produce spheroids. The different sizes determined and their distribution are presented in Figure 3, along with example images used to analyze cell volumes. In general, we found no apparent correlation between the individual cell size of a line and the resulting spheroid volume. However, there was a tendency for lines with smaller cells and a smaller range of volumes to be better suited for spheroids when higher numbers of cells are desired.

### 3.3 Distribution of living cells in a spheroid

**FIGURE 4:**
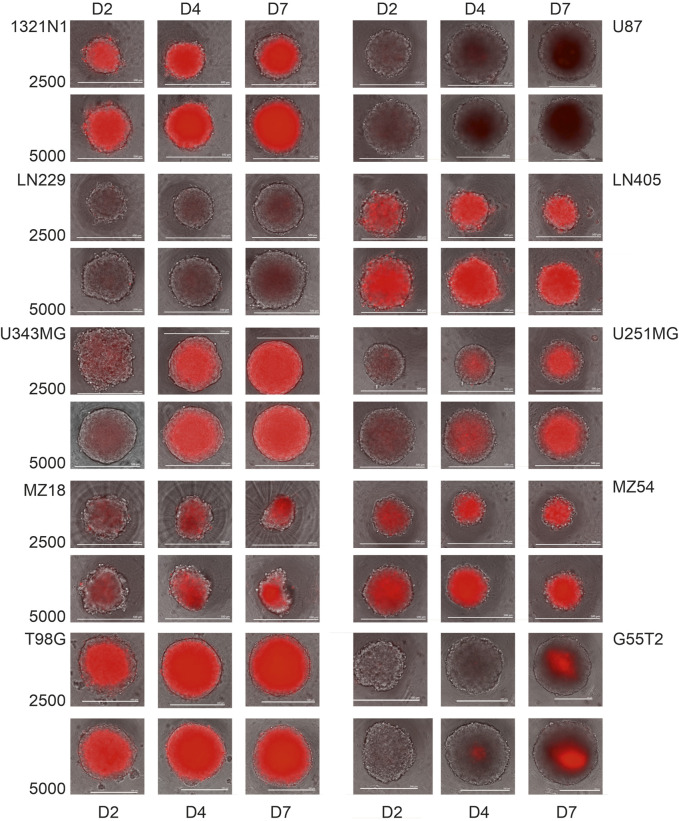
Propidium iodide staining of spheroids from the cells 1321N1, U87, LN229, LN405, U-343MG, U-251MG, MZ18 and MZ54 at 2, 4 and 7 days of growth. Cells received medium containing propidium iodide (0.1 μg/mL) before they were used to construct spheroids. Merged phase contrast and fluorescence images after 2, 4, and 7 days of cultivation are presented. Size bars: 500 µm.

As demonstrated by others, spheroids consist of an outer layer of living and proliferating cells, an intermediate layer of quiescent cells, and an inner core of dying or dead cells [e.g.,: (Sutherland et al., 1971; Onozato et al., 2017)]. To investigate whether the different lines employed in our study form spheroids with comparable cores of dead cells surrounded by living cells, we performed a microscopic analysis after staining dead cells with propidium iodide (PI). Therefore, we added PI at a 0.1 μg/mL concentration before seeding the cells for spheroid formation (2,500 and 5,000 cells; N = 5 each). The spheroids were grown for 48, 96, and 168 h and received fresh medium (50%) after 96 h. Representative images from microscopy are shown in Figure 4. As can be seen, almost all spheroids show an inner core of cells stained by PI and an outer rim of cells without staining. Staining with PI differed between the different cells; for example, cells from lines LN229 and U87 exhibited low staining at all time points, whereas a strong signal was seen in spheroids from lines such as LN405 and 1321N1. Remarkably, in some lines, staining with PI becomes more intense with increasing incubation time, indicating increasing cell death in the inner core (for example, U-251MG and U-343MG). We also tried to determine the ratio between living cells (unstained) and dead cells (stained by PI) in a spheroid. This analysis pointed towards the notion that the number of living cells in a spheroid is usually less than 10% (see Supplementary Table 2). However, as determining the border between stained and unstained cells is difficult there is a high uncertainty in this estimation.

### 3.4 Viability of spheroid and 2D cultures treated with TMZ and irradiation

**FIGURE 5:**
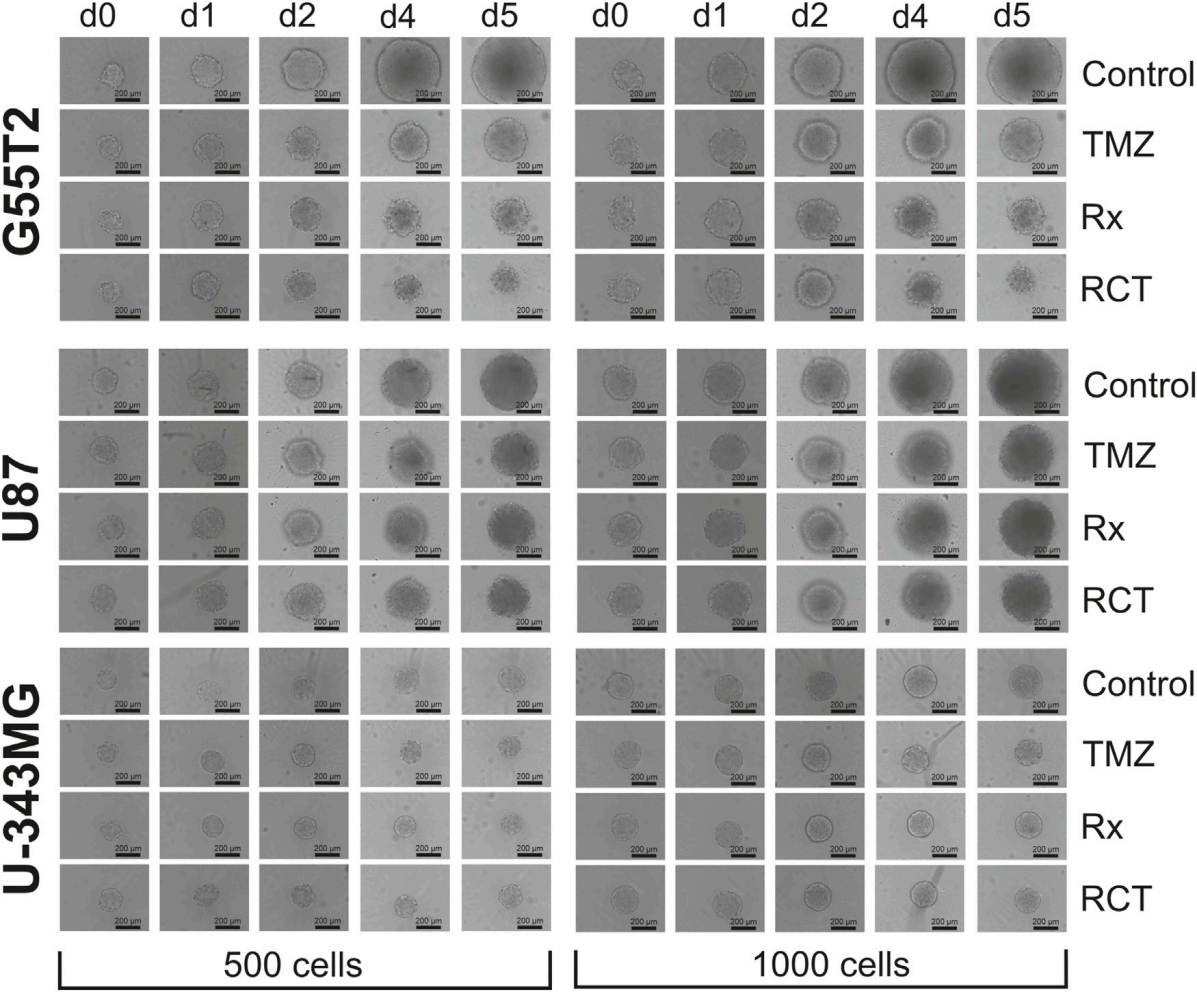
Images of spheroids after treatment with temozolomide and irradiation. Microscopic images of spheroids after treatment with temozolomide (TMZ), radiation (Rx) or by both modalities (RCT). The cells were treated 2 days after seeding for spheroid formation. d0: Spheroids just before treatment (48 h after seeding); d1, d2, d4 and d5: 1, 2, 4, and 5 days after treatment.

**FIGURE 6:**
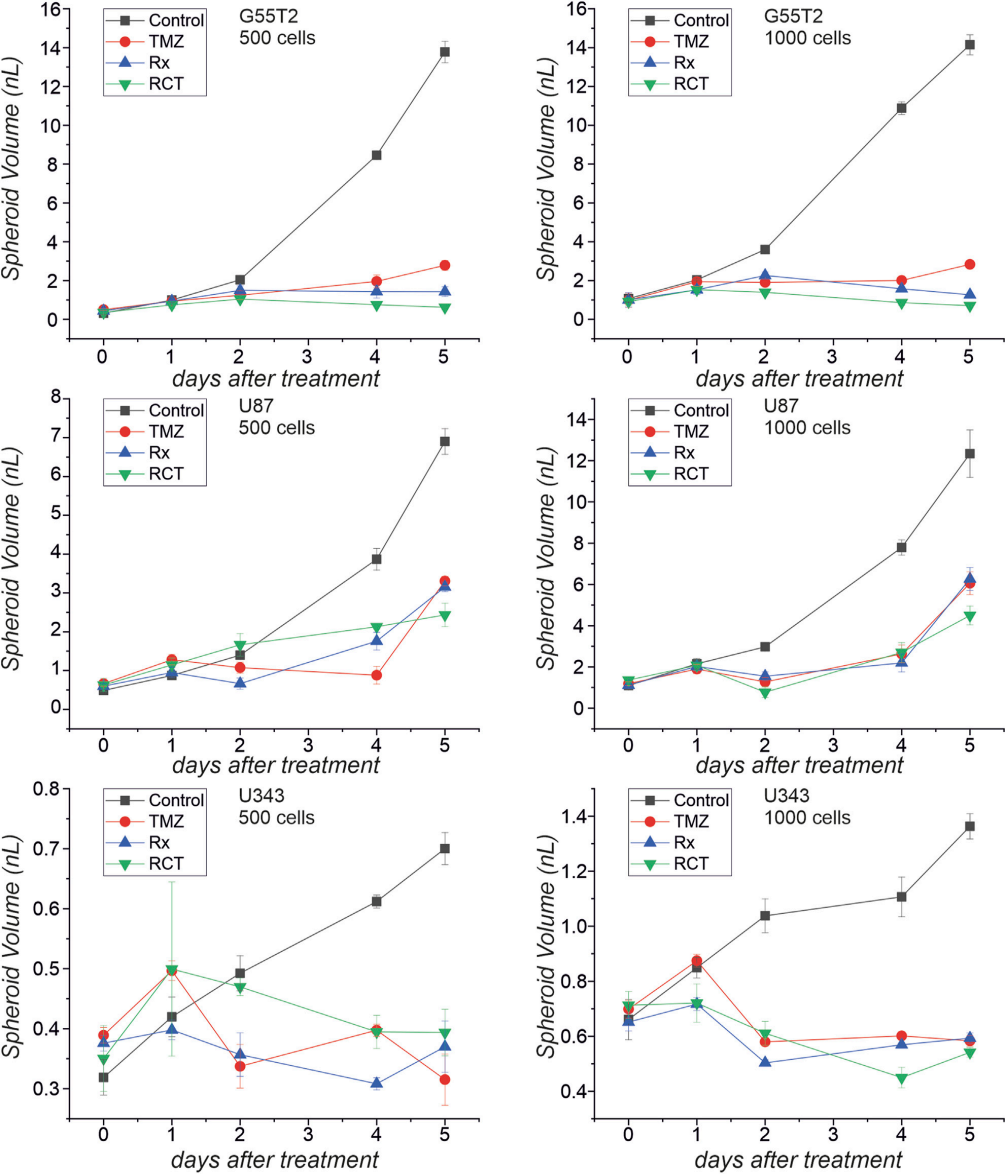
Spheroid volume after treatment with temozolomide and irradiation. The microscopic images presented in Figure 5 were used to determine spheroid volume at different times after treatment with temozolomide (TMZ), radiation (Rx) or by both modalities (RCT).

**FIGURE 7:**
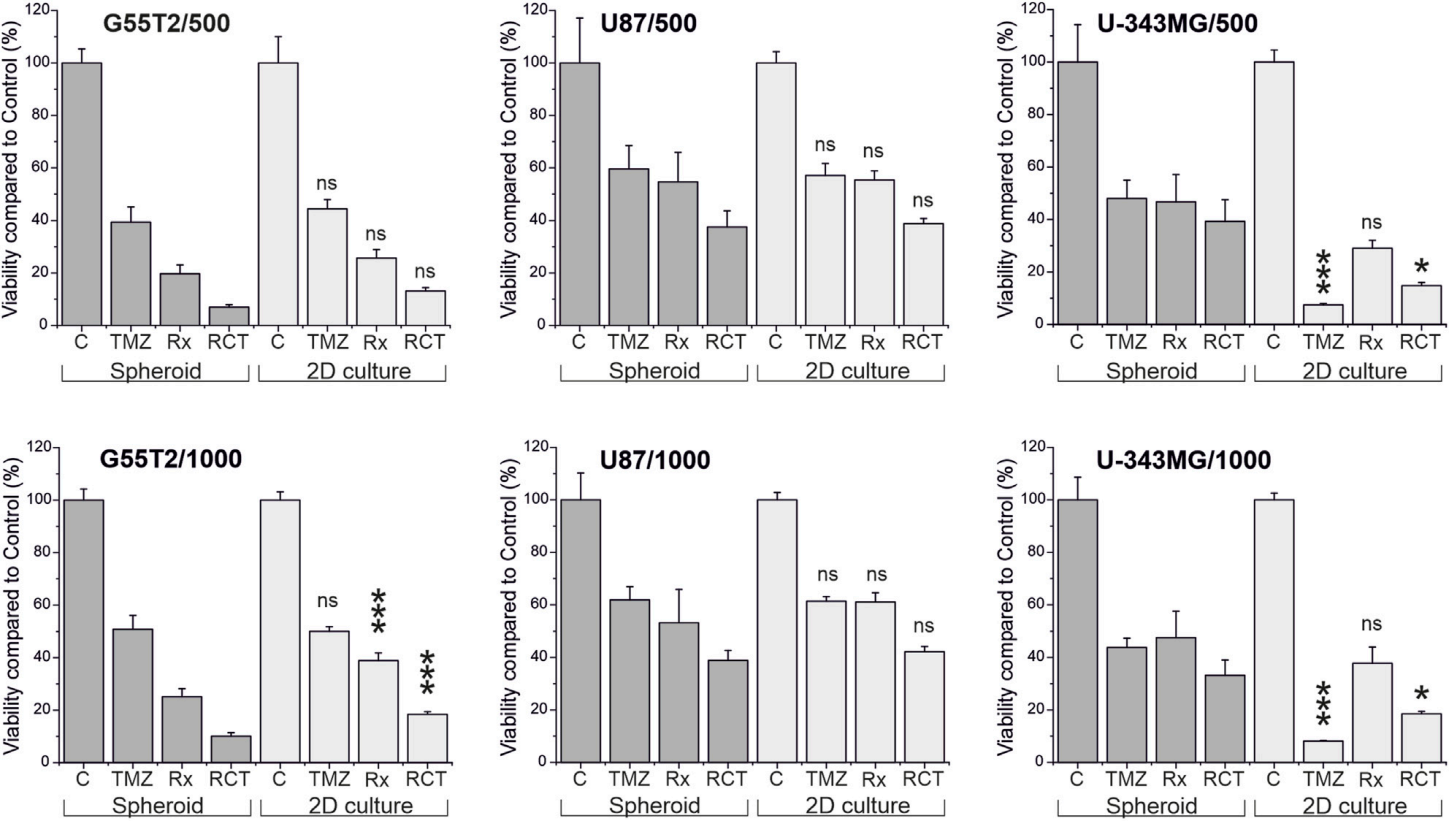
Viability of cells in 2D culture compared to viability of cells in spheroids after treatment with temozolomide and irradiation. Cultures were either irradiated (Rx), treated with temozolomide (TMZ) or received a combination of both treatments (RCT). Five days after the treatment, viability of the cultures was determined by measuring ATP in lysates using either the CellTiter Glo or the CellTiter Glo 3D assay, and the amount of ATP was compared to untreated control cultures and between the different treatment modalities. Asterisks in the graphs indicate significant differences between cells in 2D culture and spheroids (*p < 0.05; ***p < 0.0005; ns: not significant, n = 5; mean ± SD).

Finally, to determine whether spheroids respond differently to treatment than 2D cultures of the same cell line, we performed an experiment in which we treated both types of cultures with TMZ and irradiation, using cell lines G55T2, U87, and U-343MG. For this study, we created spheroids and adherent cultures using either 500 or 1,000 cells per culture well. The initial numbers were derived from Figure 2, which revealed that these lines exhibited good spheroid growth at these initial densities. After a growth period of 48 h, cells received 50 µL of fresh medium containing TMZ (200 µM final concentration) or a vehicle control without the drug. Two hours later, the cultures were irradiated (8 Gy) or sham-irradiated (0 Gy). The cells were grown for a further 5 days. During this time, selected wells were analyzed by microscopy (Figure 5). In addition, we determined the size of the spheroids of Figure 5 (data presented in Figure 6). As can be seen in Figure 6, spheroids from all lines show strong size differences at day 4 and 5. However, it should be noted that in some cases size determination was difficult because some spheroids responded to treatment with blurred edges (e.g., U87 spheroids from 1,000 cells ad day 4; Figure 5). Finally, the viability of all cultures was determined by measuring ATP in lysates after 168 h of growth (Day 5/120 h after treatment). The result of the analysis is presented in Figure 7. For the comparison, we tested whether an effect of a treatment in 2D culture is statistically different from the effect of the same treatment in 3D culture and indicated this by asterisks (e.g., *** above the column “2D culture of G55T2 cells” starting with 1,000 cells (lower left panel) indicates that the effect of irradiation alone (Rx) is significantly different (*p* > 0.0005) between 2D and 3D culture (in this case, the effect is less pronounced in 2D culture); data is also available as Supplementary Material 2 (for details of the calculation see *Materials and Methods*; “*Statistical analysis and graphical representation*”). As can be seen, cells of line U87 provide the same result regardless of the model used. The same is true for G55T2 cells when seeded at an initial density of 500 cells. In contrast, at a density of 1,000 cells, significantly stronger effects were observed in 2D culture compared to 3D culture after treatment with irradiation (Rx) or a combination of irradiation with TMZ (RCT). This different behavior could indicate that the spheroids respond more strongly to irradiation at a higher cell density.

However, it should be noted that the observed qualitative effect is the same in both models, demonstrating that irradiation has a more substantial effect on the cells than TMZ alone, and the combination of both treatment modalities has a more substantial effect than either modality alone, regardless of the initial number of cells. An even more significant difference between the responses of cells in 2D and 3D cultures can be observed for U-343MG. Here, in 2D culture, TMZ alone is more effective compared to Rx and RCT, which is not observed in 3D culture. This effect is independent of the initial number of cells used for the experiment.

## 4 Discussion

3D cultures, such as spheroids, reflect the situation in a patient’s tumor much better than the 2D cell cultures primarily used in past decades. In particular, in spheroids, only a few cells have weak contact with the material of the culture vessels and better cell-cell contacts. In addition, spheroids show gradients for oxygen, nutrients, and metabolic products typical of solid tumors (Carrasco-Mantis et al., 2023).

The critical aspect of our investigation was to analyze and compare which GBM cell lines are best suited for developing spheroids, how the spheroids grow over time, and how development, size, and growth depend on the initial number of cells employed to assemble the spheroids. Even though some comparisons have already been published, such as experiments with U-251MG, U87 and A-172 (Wanigasekara et al., 2023), and there is data from other cell lines [For instance, SNB19 (Nirmala et al., 2001), T98G (Oraiopoulou et al., 2019), LN229 (Singh et al., 2020)], a broader systematic comparison of cells from different lines is missing. It should also be noted that many methods are employed throughout the literature. In addition, conditions of cultivation vary between different approaches. For instance, Nirmala and colleagues cultured their spheroids made from the glioblastoma line SNB19 on an agar substrate and monitored growth over up to 30 days (Nirmala et al., 2001), and Alves et al. (2023), who used cells from the lines U87, T98G, UW473, A172 and U-251MG, used type I collagen as an extracellular matrix during spheroid formation. It is beyond the scope of our manuscript to compare the different methods used in the literature. Instead, we focused on the development of spheroids in ULA plates as it is considered that this method is best suited to produce a large number of comparable spheroids in a short period (Habra et al., 2023) [for a discussion of other methods, see (Nath and Devi, 2016)]. At this point, we would also like to recommend the manuscript of Wanigasekara and coworkers, who compared different methods for spheroid formation using cells from the GBM lines U87, U-251MG and A-172 (Wanigasekara et al., 2023).

The first question we addressed was the spheroid size and development dependence on the initial number of cells using different lines. Therefore, we determined the volumes of the spheroids by a relatively simplistic method assuming an almost round-shaped appearance, which appeared to be a good estimation to compare the different sizes obtained using different amounts of cells and different incubation times. However, one should be aware that the exact volume of the spheroids may be slightly different. Therefore, if research requires a precise determination of volumes instead of an estimation, refer to an alternative algorithm or method (Zanoni et al., 2016).

First of all, our experiments revealed that although spheroids were obtained from all cell lines, spheroids built from lines LN405, MZ18, and MZ54 did not grow. We did not identify any publication presenting data on spheroids comprising these GBM cell lines. Even though all other lines could build growing spheroids when initial densities of up to 500 cells were used, some spheroids did not grow at higher cell numbers. For instance, cells from line U-251MG even exhibited a decreasing size with prolonged incubation time when more than 500 cells were used. Other cells also revealed significant growth in 1,000 and 2,000 cells. However, using cell numbers of 5,000 or 15,000 cells did not result in significantly growing spheroids. All our cells have been cultivated in the same medium (DMEM with 4.5 g/L glucose, 10% FCS, and 2 mM GlutaMAX), which has been tested and used for 2D culture in our group for years (See Supplementary Table 3 for cell doubling times in 2D culture). However, we cannot rule out that some cells may require a different medium for healthy growth in 3D culture. At this point, we also have to remark that Wanigasekara et al. reported spheroid growth between 48 and 96 h when using U-251MG cells at an initial density of 2,000 cells (10,000 cells/mL) (Wanigasekara et al., 2023), using quite similar conditions to ours. Therefore, we need to determine whether this deviation from our observation is caused by differences in the medium or the use of culture plates from a different manufacturer. However, this observation stresses that individual testing of the appropriate number of cells for spheroid formation should be performed before extensive experiments are initiated, as there might be parameters affecting the outcome of yet unknown origin and depending on the specific setup in the lab.

Generally, spheroids appear to be compact with an inner core of dead cells, as seen through propidium iodide staining, which is a well-known phenomenon [e.g., (Eilenberger et al., 2018; Singh et al., 2020; Phour and Vassella, 2024) and others]. Unfortunately, we did not perform life-cell staining, e.g., by Calcein-AM, which could have helped to determine the exact size of the rim of living cells. In further experiments, this issue may be resolved by staining spheroids at appropriate time points and immediate imaging, as done by Singh et al. (2020). An alternative high-resolution tomographic analysis may also be considered (Ozturk et al., 2020). (Note: We did not consider FACS analysis after disruption of spheroids into single cells because of the highly necrotic core, which we expected to lose dead and dying cells during disruption and preparation for FACS, and therefore to misleading counts). Interestingly, just recently, Phour and Vassella presented a protocol using life cell imaging and staining by Calcein-AM, Helix NP, which appears to be a highly reliable method for the determination of living and dead cells in a spheroid (Phour and Vassella, 2024), and which can be used to determine the exact ratio of living and dead cells, and could also be an option for the determination of drug efficacy in toxicity studies. The same holds for the use of alamarBlue, which may also be an alternative for the determination of the viability of the spheroids, especially for drug testing and toxicity studies (Eilenberger et al., 2018).

When comparing spheroids with 2D cultures after treatment with TMZ and irradiation, we observed a similar response from cells of line U87 but differences in lines G55T2 and U-343MG, with a tendency towards a less pronounced response in spheroid culture. For these experiments, we used a 3D assay (CellTiter Glo 3D), which is based on the reaction of luciferin to oxyluciferin in the presence of ATP derived from living cells. Unfortunately, the spheroids are lysed before analysis, resulting in an end-point measurement, and prolonged live-cell imaging is impossible with this technique. This problem may be resolved by live cell imaging, as it has been done by Kessel et al. using spheroids from U87 cells and Celigo Image Cytometry (Kessel et al., 2017). Furthermore, the methods discussed in the previous section may be considered, especially the method introduced by Phour and Vassella (Phour and Vassella, 2024). In general, it has to be noted that the robustness of drug testing is deeply dependent on the assays employed for measuring the toxicity of a drug. As this was not the intention of our current research, further research is required to evaluate the robustness of viability assays, considering HTS approaches using ULA plates and GBM cell lines according to the protocols presented in this manuscript.

Compared to other human 3D glioblastoma models, such as tumor spheres, organotypic slices, explants, tumoroids, or glioblastoma-derived cerebral organoids [for a review, see (Soubéran and Tchoghandjian, 2020)], spheroids are a relatively simple model with some limitations. The main limitation is that the model does not reflect the heterogeneity typical of glioblastomas. This aspect may be considered by forming spheroids from patient-derived glioblastoma cells (Brown et al., 2023; Yuzhakova et al., 2023). However, these models are also limited, as each culture is unique for the patient from which it was derived, limiting reproducibility by other groups. In addition, it has to be taken into account that cell cultures need to be propagated after they have been established in order to have enough cells, in case one is interested in testing different concentrations of a substance or substance combinations. Propagation, on the other hand, may cause changes in the relation of different cell types in the culture with each passage or may lead to genetic alterations.

On the other hand, as demonstrated by Nickel et al., at least tumor spheroid cell lines appear to be genetically stable over prolonged culture times (30 *in vitro* passages) (Nickel et al., 2021). Another interesting approach to simulate heterogeneity in a spheroid model with well-characterized cells is proposed by Sivakumar and coworkers (Sivakumar et al., 2020). This group used up to 4 GBM cell lines and one astrocyte line, where the cells of each line were labeled with different fluorescent dyes, and the spheroids were embedded in a brain-like hyaluronic acid hydrogel. Another approach could be using GBM cell lines together with astrocytes (UP-010), which was successfully done by Civita et al. (2019). However, the additional treatments required also mean the experimental effort increases accordingly. Given that spheroids generated from different GBM lines can also react differently to treatment, the question should be asked whether the lack of heterogeneity within a spheroid could be compensated for using more than one cell line to form spheroids during substance testing.

## 5 Conclusion

The production of spheroids using ULA plates is a rapid and cost-effective method that allows the production of high numbers of spheroids, which facilitates extensive screening of various substances, concentrations, and combinations. Here, we demonstrate that in advance of pursuing a more extensive series of experiments, e.g., HTS, one should carefully optimize culture conditions, especially regarding initial cell number, growth behavior of spheroids, and choice of cell lines. Our data is a valuable starting point for the initiation of stringent drug toxicity testing using spheroids derived from cell lines.

## Data Availability

The original contributions presented in the study are included in the article/Supplementary Material, further inquiries can be directed to the corresponding author.
