# Supplementary material for: Rapid and reproducible generation of glioblastoma spheroids for high-throughput drug screening
Source: Front Bioeng Biotechnol. 2024 Dec 18;12:1471012. doi: 10.3389/fbioe.2024.1471012 (PMC11688379; doi:10.3389/fbioe.2024.1471012)
Supplement: Supplementary file 1 [file DataSheet2.pdf]

Supplement 2 Data of graphical image presented in Figure 7

The data presented in this supplement ist the data used for the presentation Figure 7 (Viability of cells in 2D culture compared to viability of cells in spheroids after treatment with temozolomide and irradiation). Cultures were either irradiated (Rx), treated with temozolomide (TMZ) or received a combination of both treatments (RCT). Five days after the treatment, viability of the cultures was determined by measuring ATP in lysates using either the CellTiter Glo or the CellTiter Glo 3D assay, and the amount of ATP was compared to untreated control cultures and between the different treatment modalities.

Viability of 2D and 3D culture after treatment with temozolomide (TMZ), irradiation (Rx) or both modalities (RCT)

		2D Culture		Spheroids	
		Mean (%)	E (%)	Mean (%)	E (%)
G55T2 500 cells	Control	100,00	10,02	100,00	5,33
	TMZ	44,40	3,56	39,37	5,73
	Rx	25,68	3,28	19,73	3,35
	RCT	13,16	1,24	6,96	0,90

		2D Culture		Spheroids	
		Mean (%)	E (%)	Mean (%)	E (%)
G55T2 1000 cells	Control	100,00	3,15	100,00	4,23
	TMZ	50,06	1,76	50,82	5,22
	Rx	38,86	2,94	25,10	3,08
	RCT	18,41	0,98	10,08	1,32

		2D Culture		Spheroids	
		Mean (%)	E (%)	Mean (%)	E (%)
U87 500 cells	Control	100,00	4,30	100,00	17,14
	TMZ	57,13	4,52	59,63	8,84
	Rx	55,43	3,46	54,67	11,25
	RCT	38,78	2,03	37,54	6,10

		2D Culture		Spheroids	
		Mean (%)	E (%)	Mean (%)	E (%)
U87 1000 cells	Control	100,00	2,81	100,00	10,24
	TMZ	61,41	1,73	61,86	5,04
	Rx	61,05	3,56	53,15	12,67
	RCT	42,14	2,04	38,85	3,80

		2D Culture		Spheroids	
		Mean (%)	E (%)	Mean (%)	E (%)
U343 500 cells	Control	100,00	4,60	100,00	14,26
	TMZ	7,47	0,58	48,00	6,98
	Rx	29,06	3,04	46,72	10,34
	RCT	14,81	1,23	39,29	8,25

		2D Culture		Spheroids	
		Mean (%)	E (%)	Mean (%)	E (%)
U343 1000 cells	Control	100,00	2,57	100,00	8,62
	TMZ	8,12	0,25	43,77	3,57
	Rx	37,79	6,16	47,55	9,97
	RCT	18,49	0,88	33,20	5,83
