# Supplementary material for: Rapid and reproducible generation of glioblastoma spheroids for high-throughput drug screening
Source: Front Bioeng Biotechnol. 2024 Dec 18;12:1471012. doi: 10.3389/fbioe.2024.1471012 (PMC11688379; doi:10.3389/fbioe.2024.1471012)
Supplement: Supplementary file 2 [file DataSheet4.pdf]

Supplemental Table 2. Ratio between Unstained cells and cells stained by PI in a spheroid

Spheroids from 10 different GBM cell lines were grown for 2, 4 and 7 days starting either with 2500 or with 5000 cells (n=5 spheroids for each condition; each in a separate well). Cells received Propidium Iodide (PI; 0.1 µg/ml) the day, they were seeded. At the days indicated, the total size of the spheroids was determined after phase contrast microscopy and the volume of the inner core of cells stained by PI was determined after fluorescence microscopy. In some cases, it was not possible to determine the volume of the spheroid or the stained core, because the spheroids deviated too much from a sphere. In addition, in some cases the border between stained and unstained cells could not be identified with certainty (though we used different exposure times for fluorescence microscopy). Therefore, in some cases, it was not possible to calculate the ratio between the volumes of the inner core and the total size of the spheroid and "n" was not in any case 5 (the number of spheroids originally formed).

Cell line	Initial amount of cells	Unstained Cells percent		
		48 hours	96 hours	168 hours
U-251MG	2500	5.87 ± 0.66	3.80 ± 0.25	3.98 ± 0.23
	5000	2.16 ± 0.12	1.69 ± 0.08	4.77 ± 0.34
1321N1	2500	4.28 ± 0.40	2.10 ± 0.13	3.91 ± 0.28
	5000	1.45 ± 0.06	0.63 ± 0.03	1.24 ± 0.04
LN405	2500	3.78 ± 0.39	1.25 ± 0.09	1.74 ± 0.13
	5000	2.09 ± 0.11	0.46 ± 0.02	0.88 ± 0.04
MZ18	2500	2.21 ± 0.10	0.48 ± 0.01	1.68 ± 0.11
	5000	6.87 ± 0.99	1.81 ± 0.25	5.34 ± 1.02
T98G	2500	5.87 ± 0.59	1.33 ± 0.07	1.43 ± 0.05
	5000	5.20 ± 0.63	1.23 ± 0.10	2.77 ± 0.19
MZ54	2500	3.86 ± 0.33	1.97 ± 0.18	2.49 ± 0.17
	5000	1.95 ± 0.08	1.53 ± 0.08	2.22 ± 0.10
U-343MG	2500	3.67 ± 0.32	0.31 ± 0.01	1.22 ± 0.05
	5000	3.41 ± 0.46	1.11 ± 0.10	2.15 ± 0.14
G55T2	2500			6.04 ± 0.42
	5000		4.23 ± 0.20	7.70 ± 0.70
LN229	2500			3.96 ± 0.22
	5000			3.88 ± 0.22
