# Supplementary material for: Rapid and reproducible generation of glioblastoma spheroids for high-throughput drug screening
Source: Front Bioeng Biotechnol. 2024 Dec 18;12:1471012. doi: 10.3389/fbioe.2024.1471012 (PMC11688379; doi:10.3389/fbioe.2024.1471012)
Supplement: Supplementary file 3 [file DataSheet3.pdf]

Supplemental Table 1. Linear regression curves of data presented in Figure 1.

The data presented in Figure 1 was used to perform a linear regression by fitting the non-logarithmic data to the formula y = a + b*x with y: volume of spheroid; a: intercept; b: slope and x: number of cells.

U87	Intercept (Volume/nL)		Slope (pL / Zellnumber)		
	Value	SE	Value	SE	adj. R^2
24 h	-1,65	1,03	7,81	0,16	0,99796
48 h	3,20	1,60	8,49	0,25	0,99581
72 h	8,83	3,63	9,36	0,56	0,98255
96 h	15,88	5,22	10,41	0,80	0,97115
120 h	34,17	10,65	12,47	1,63	0,91975
144 h	56,41	18,04	13,94	2,77	0,82983
168 h	69,32	19,13	15,17	2,93	0,83751

U-251MG	Intercept (Volume/nL)		Slope (pL / Zellnumber)		
	Value	SE	Value	SE	adj. R^2
24 h	-6,56	4,32	12,23	0,66	0,9855
48 h	-2,70	1,99	9,24	0,30	0,99458
72 h	-2,16	1,95	8,09	0,30	0,99318
96 h	-1,18	1,65	7,17	0,25	0,99381
120 h	-0,19	1,10	6,59	0,17	0,99671
144 h	0,48	1,02	6,40	0,16	0,99703
168 h	0,70	0,92	6,29	0,14	0,9975

G55T2	Intercept (Volume/nL)		Slope (pL / Zellnumber)		
	Value	SE	Value	SE	adj. R^2
24 h	-3,49	1,95	6,84	0,30	0,99049
48 h	0,24	0,41	8,08	0,06	0,9997
72 h	9,10	4,37	11,06	0,67	0,98194
96 h	24,26	9,75	12,58	1,49	0,93321
120 h	53,67	14,96	13,99	2,29	0,87858
144 h	89,48	18,76	15,16	2,88	0,84264
168 h	115,04	19,57	16,42	3,00	0,85283

U-343MG	Intercept (Volume/nL)		Slope (pL / Zellnumber)		
	Value	SE	Value	SE	adj. R^2
24 h	-7,40	2,97	13,78	0,45	0,99457
48 h	-1,81	1,30	7,82	0,20	0,99675
72 h	-0,48	0,79	6,93	0,12	0,99847
96 h	3,87	1,39	6,01	0,21	0,99372
120 h	5,34	2,03	6,48	0,31	0,98862
144 h	6,89	2,26	6,95	0,35	0,98772
168 h	10,00	3,18	7,14	0,49	0,9771

T98G	Intercept (Volume/nL)		Slope (pL / Zellnumber)		
	Value	SE	Value	SE	adj. R^2
24 h	-8,04	5,00	18,98	0,77	0,99191
48 h	1,58	1,39	13,89	0,21	0,99882
72 h	11,98	3,91	11,63	0,60	0,98684
96 h	20,39	6,07	10,18	0,93	0,95966
120 h	28,50	6,62	9,41	1,01	0,94449
144 h	37,04	5,37	10,41	0,82	0,96946
168 h	46,95	5,55	9,72	0,85	0,96286

LN405	Intercept (Volume/nL)		Slope (pL / Zellnumber)		
	Value	SE	Value	SE	adj. R^2
24 h	-3,53	2,92	14,85	0,45	0,99546
48 h	-3,23	2,68	9,84	0,41	0,99135
72 h	-1,97	1,40	7,55	0,21	0,996
96 h	-1,36	0,58	6,55	0,09	0,99907
120 h	-1,08	0,77	5,73	0,12	0,9979
144 h	-0,67	0,46	5,24	0,07	0,99911
168 h	-1,70	1,05	4,84	0,16	0,99445

LN229	Intercept (Volume/nL)		Slope (pL / Zellnumber)		
	Value	SE	Value	SE	adj. R^2
24 h	-3,93	1,80	8,05	0,28	0,99417
48 h	-1,12	0,90	5,64	0,14	0,99702
72 h	0,63	0,42	5,06	0,06	0,99919
96 h	2,42	0,78	4,97	0,12	0,99711
120 h	4,17	1,02	5,27	0,16	0,99562
144 h	7,61	2,28	5,44	0,35	0,97963
168 h	10,63	3,28	5,38	0,50	0,95793

MZ18	Intercept (Volume/nL)		Slope (pL / Zellnumber)		
	Value	SE	Value	SE	adj. R^2
24 h	6,13	3,50	7,52	0,54	0,97498
48 h	3,35	1,16	5,42	0,18	0,99464
72 h	2,52	0,55	4,41	0,08	0,99819
96 h	2,67	0,98	3,12	0,15	0,9885
120 h	2,53	0,71	2,86	0,11	0,99285
144 h	2,15	0,52	2,28	0,08	0,99383
168 h	1,93	0,53	1,86	0,08	0,9906

1321N1	Intercept (Volume/nL)		Slope (pL / Zellnumber)		
	Value	SE	Value	SE	adj. R^2
24 h	-3,30	1,89	8,49	0,29	0,99417
48 h	-0,46	0,85	6,00	0,13	0,99763
72 h	1,45	0,93	5,83	0,14	0,997
96 h	4,35	0,72	5,16	0,11	0,99769
120 h	8,64	1,90	5,80	0,29	0,98756
144 h	12,91	2,93	6,29	0,45	0,97502
168 h	18,16	4,35	6,07	0,67	0,94242

MZ54	Intercept (Volume/nL)		Slope (pL / Zellnumber)		
	Value	SE	Value	SE	adj. R^2
24 h	-4,36	1,69	10,36	0,26	0,99687
48 h	-0,37	0,36	4,58	0,06	0,99926
72 h	0,56	0,06	3,24	0,01	0,99996
96 h	0,82	0,08	2,33	0,01	0,99986
120 h	0,80	0,11	1,86	0,02	0,99956
144 h	1,19	0,14	1,58	0,02	0,99903
168 h	1,25	0,27	1,42	0,04	0,99587
