# Supplementary material for: Rapid and reproducible generation of glioblastoma spheroids for high-throughput drug screening
Source: Front Bioeng Biotechnol. 2024 Dec 18;12:1471012. doi: 10.3389/fbioe.2024.1471012 (PMC11688379; doi:10.3389/fbioe.2024.1471012)
Supplement: Supplementary file 4 [file DataSheet1.pdf]

Supplement 1

The panels show microscopic images of spheroids built from different initial numbers of cells (indicated at the left) at 1 to 7 days of cultivation (indicated on top). The images were obtained by microscopy with a Keyence BZ-X800 microscope and were constructed from individual pictures stitched by the Keyence analyzer software in order to demonstrate the complete spheroids. All size bars are 500 µm.

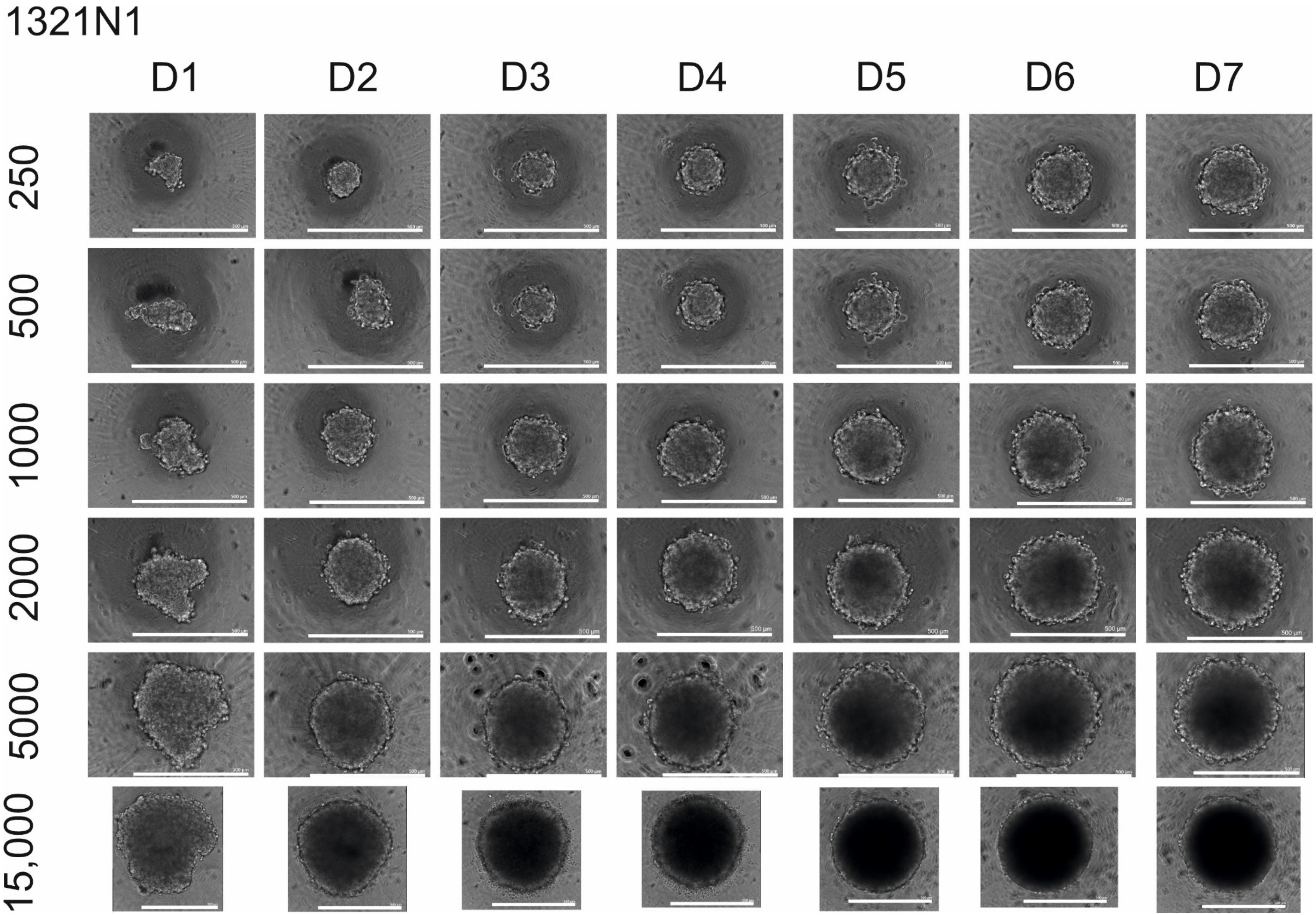
1321N1
D1
D2
D3
D4
D5
D6
D7
250
500
1000
2000
5000
15,000

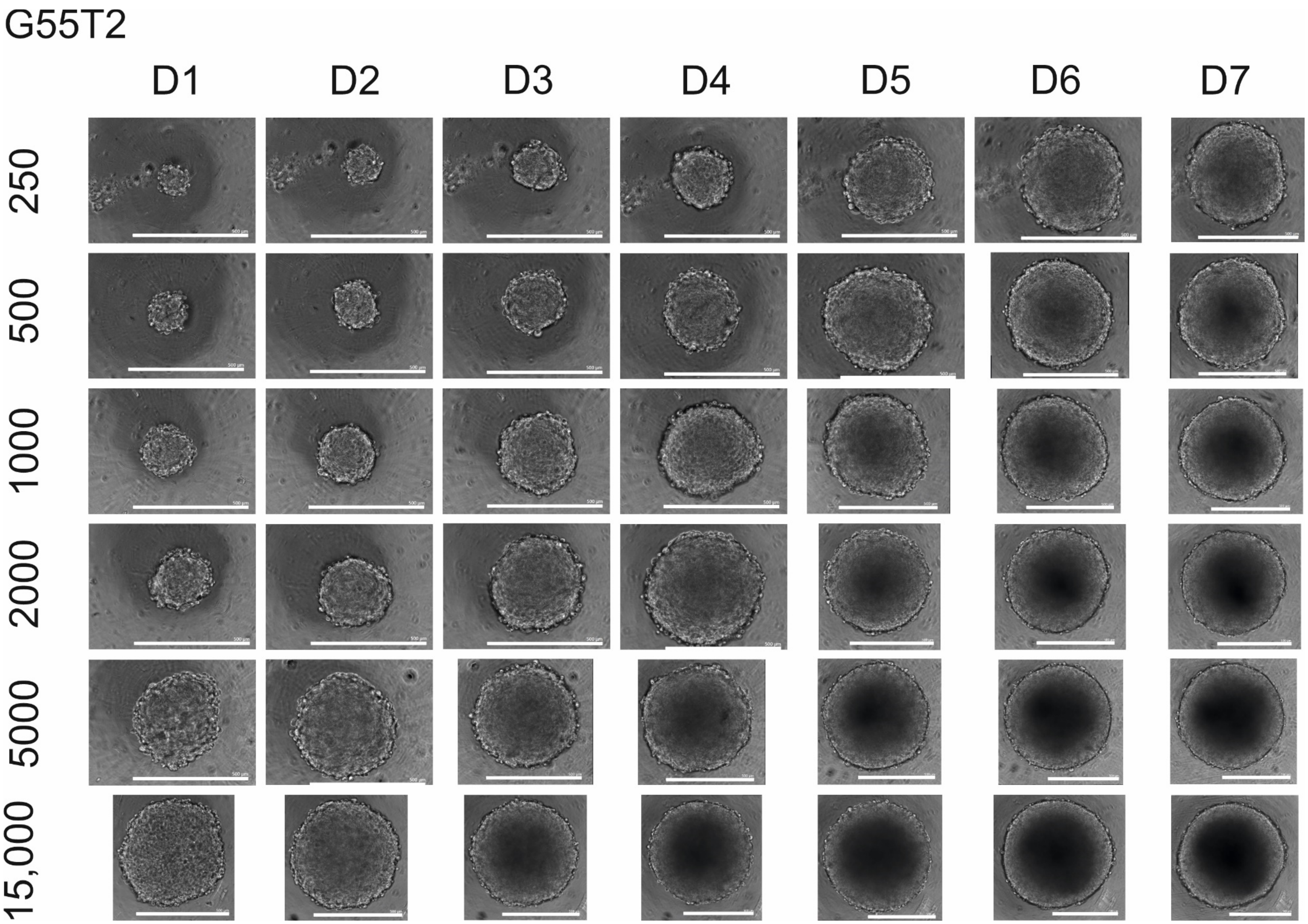
G55T2
D1
D2
D3
D4
D5
D6
D7
250
500
1000
2000
5000
15,000

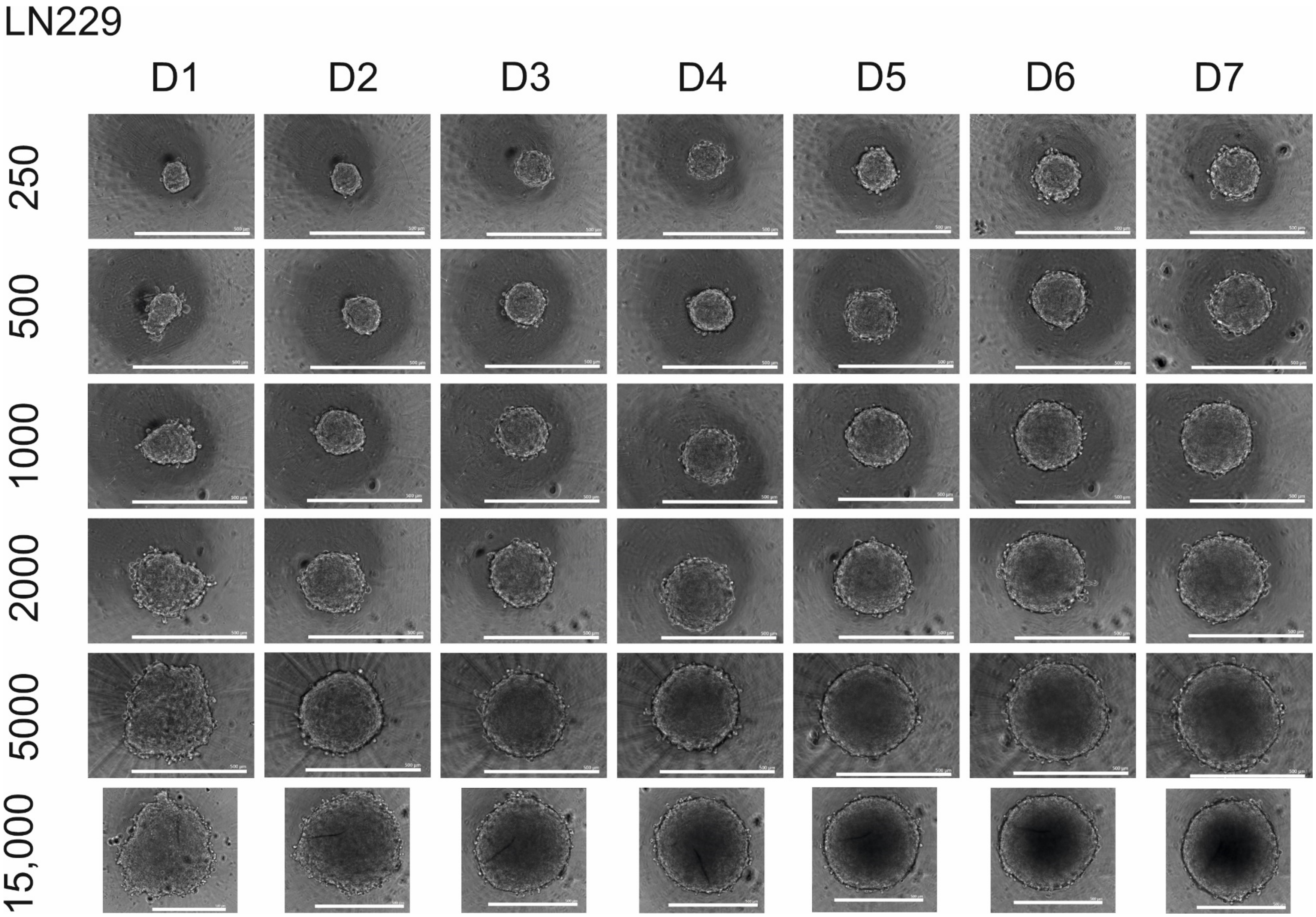
LN229
D1
D2
D3
D4
D5
D6
D7
250
500
1000
2000
5000
15,000

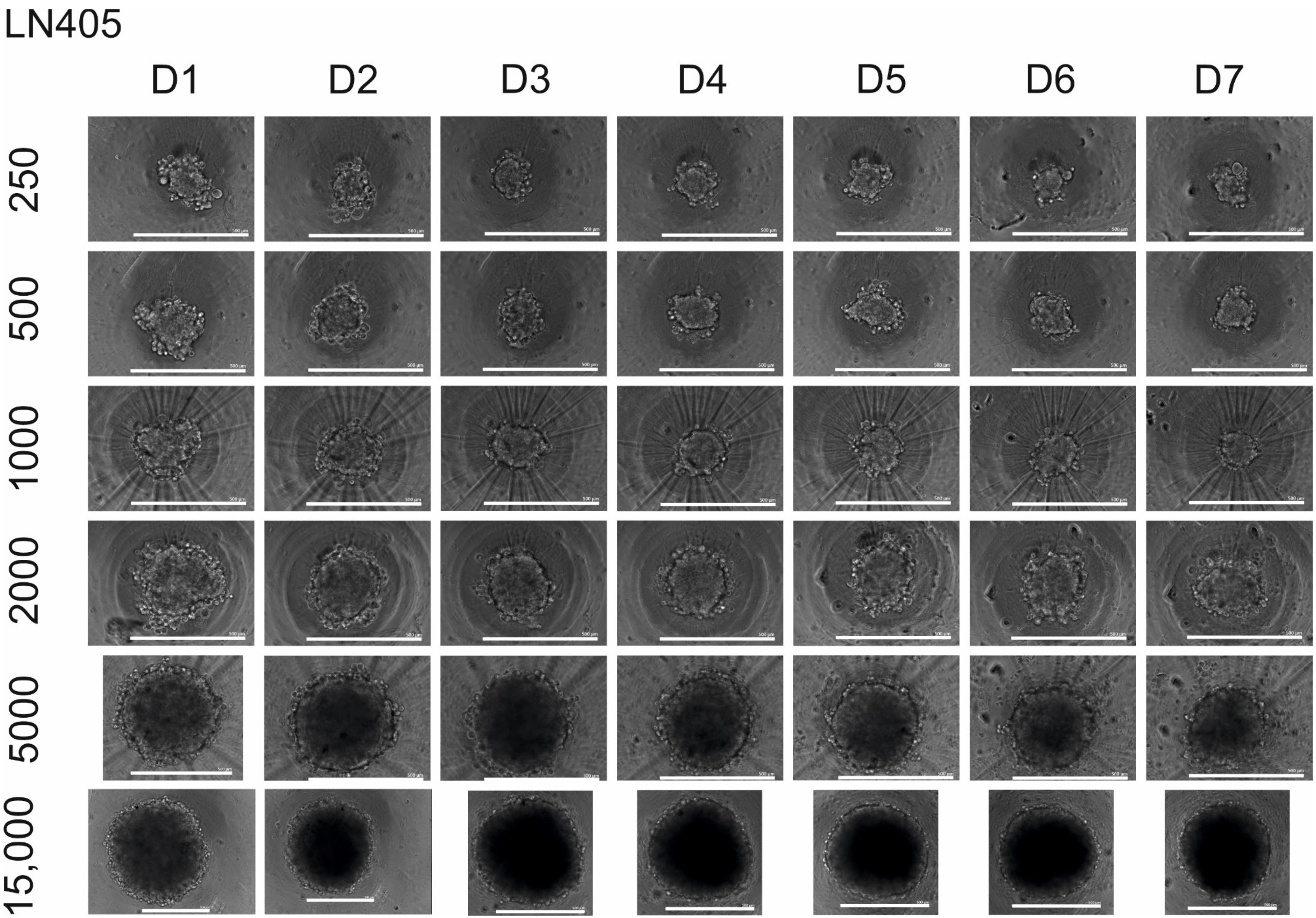

LN405
D1
D2
D3
D4
D5
D6
D7
250
500
1000
2000
5000
15,000

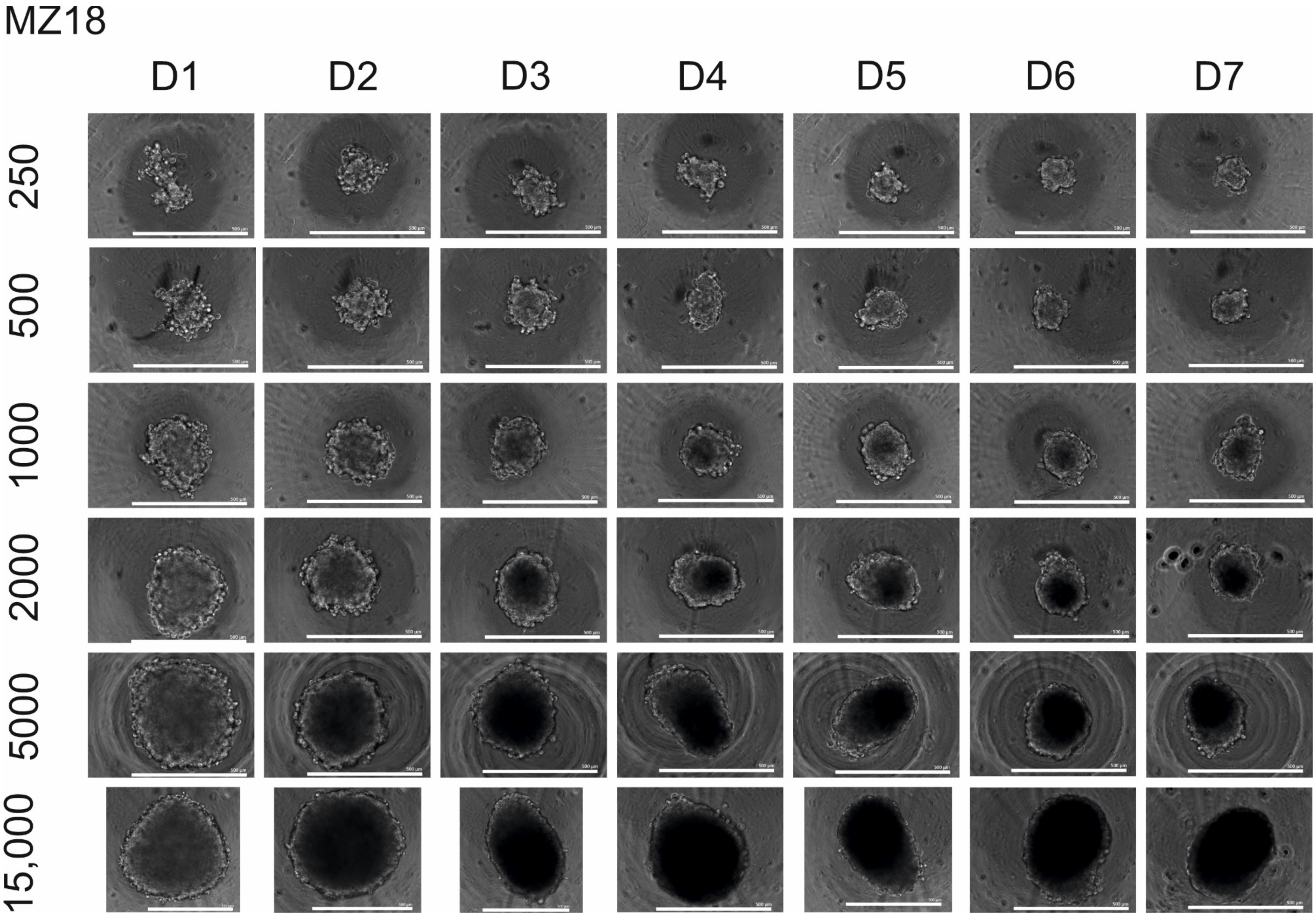
MZ18
D1
D2
D3
D4
D5
D6
D7
250
500
1000
2000
5000
15,000

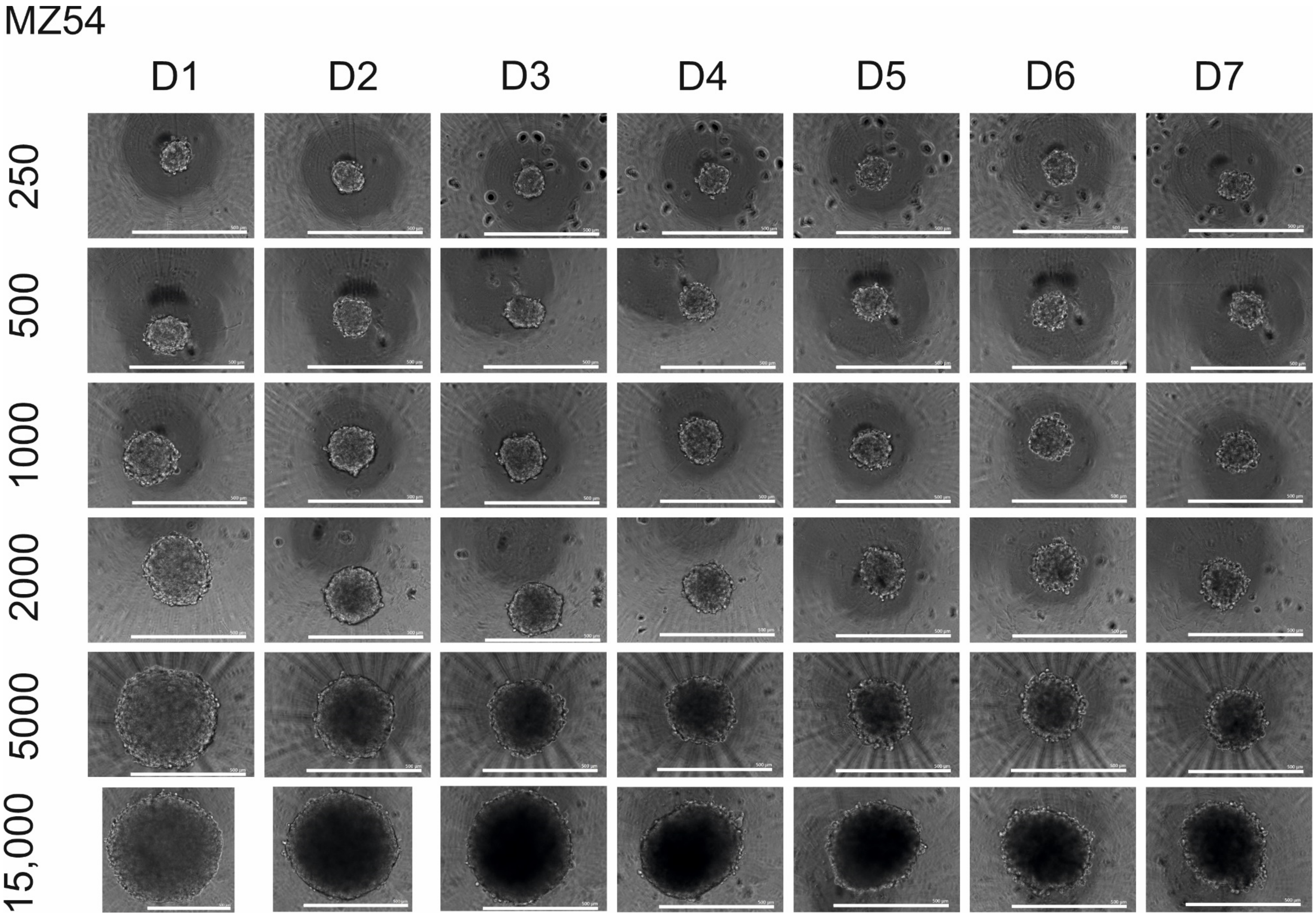
MZ54
D1
D2
D3
D4
D5
D6
D7
250
500
1000
2000
5000
15,000

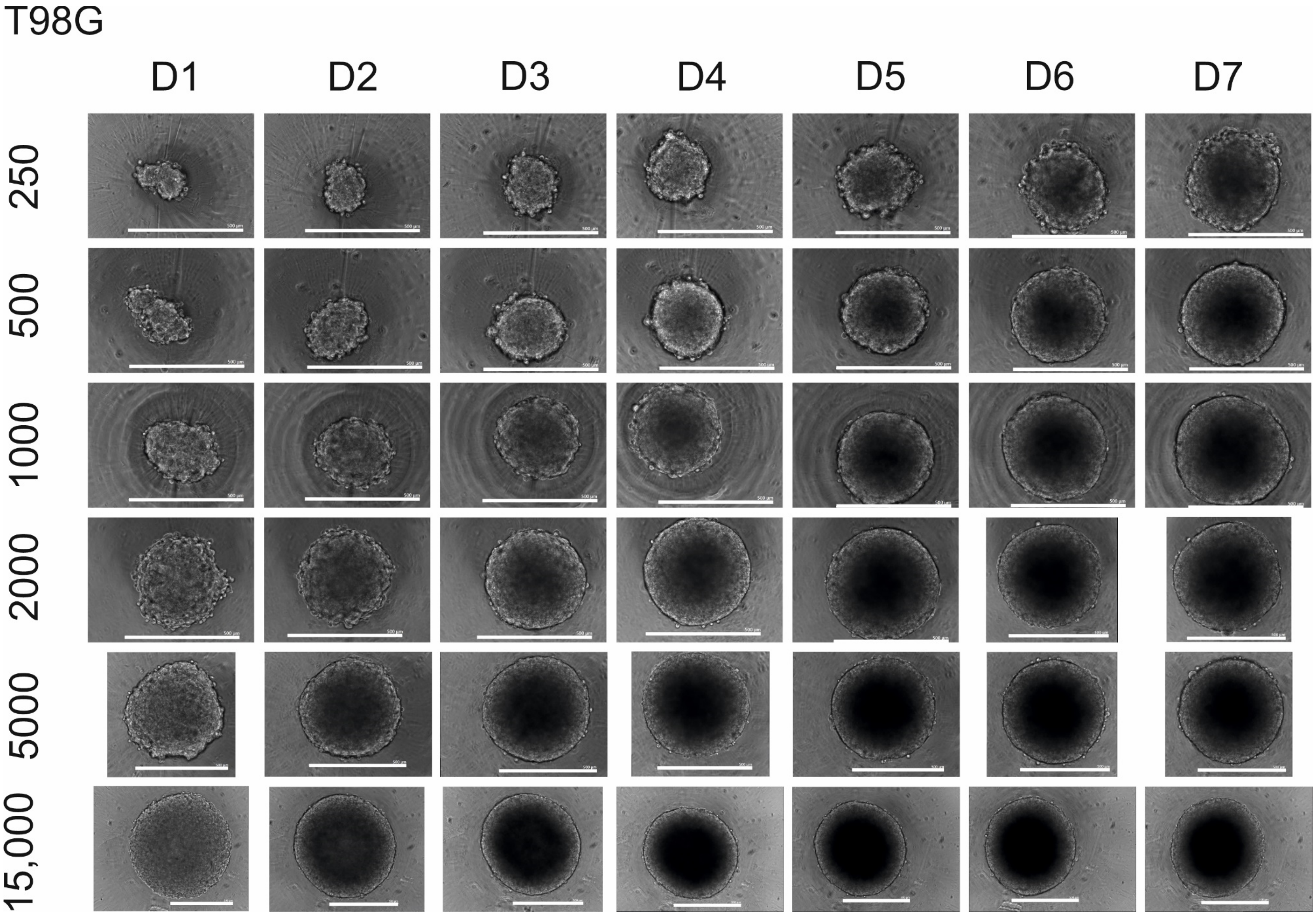
T98G
D1
D2
D3
D4
D5
D6
D7
250
500
1000
2000
5000
15,000

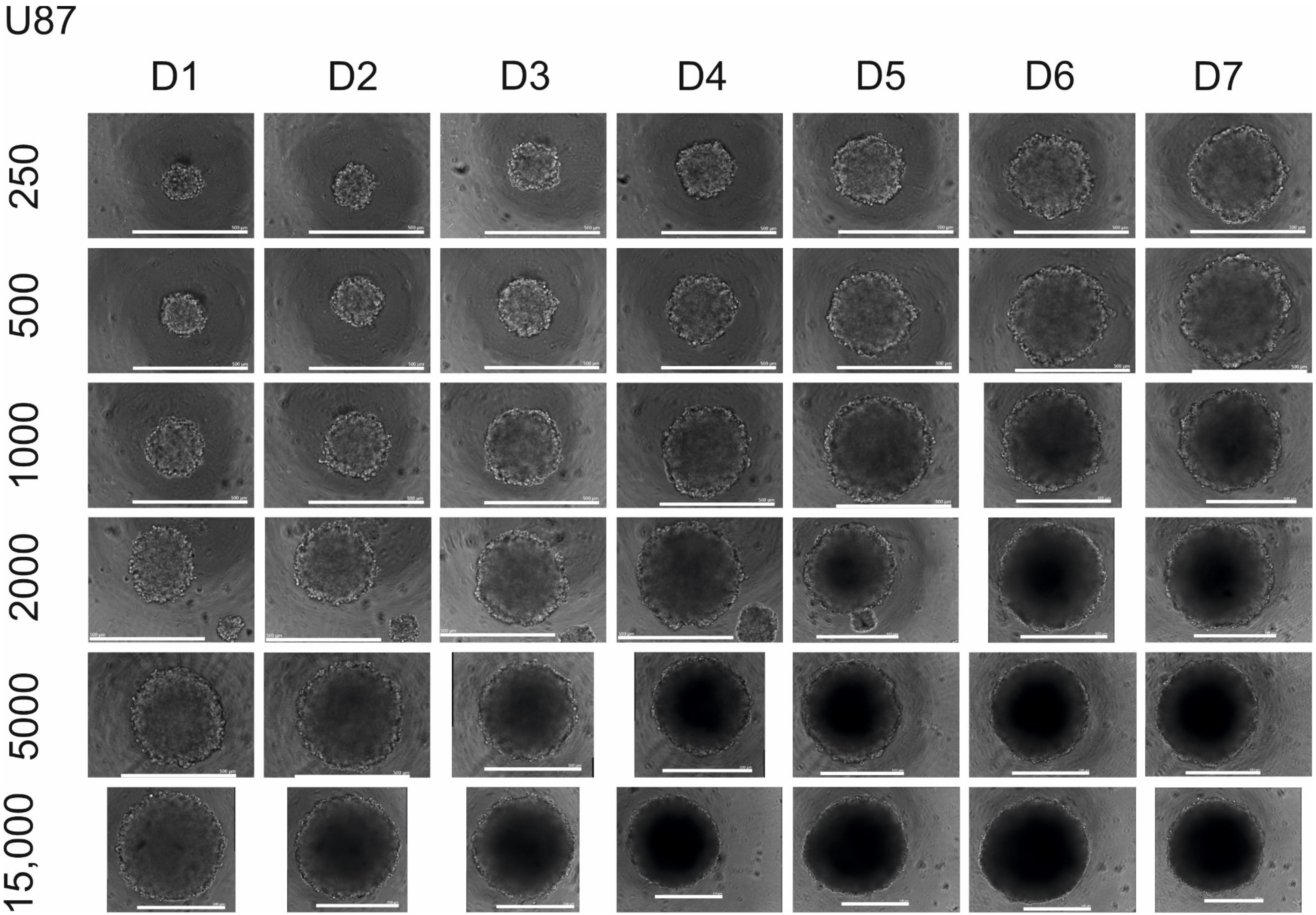

U87
D1
D2
D3
D4
D5
D6
D7
250
500
1000
2000
5000
15,000

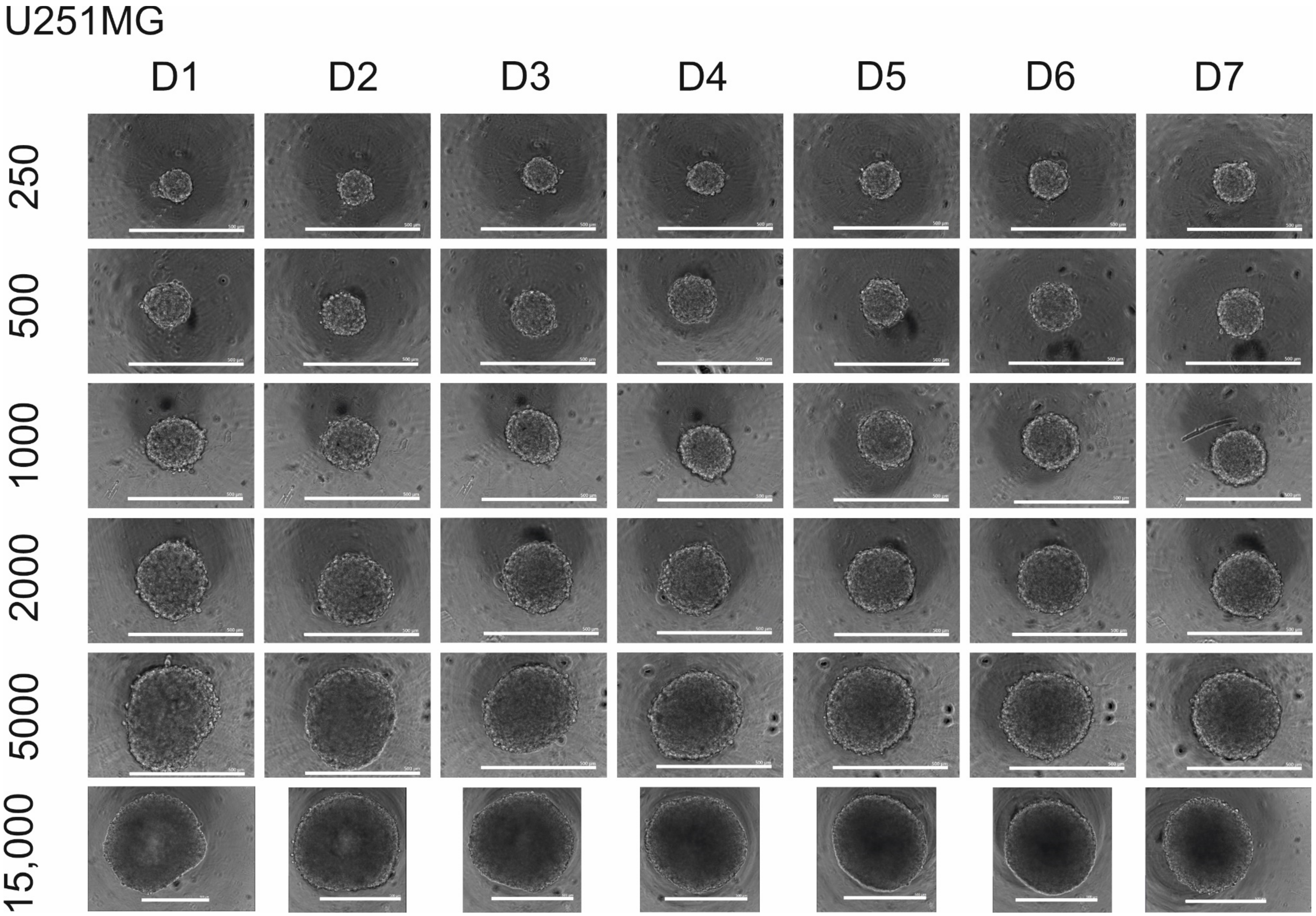
U251MG
D1
D2
D3
D4
D5
D6
D7
250
500
1000
2000
5000
15,000

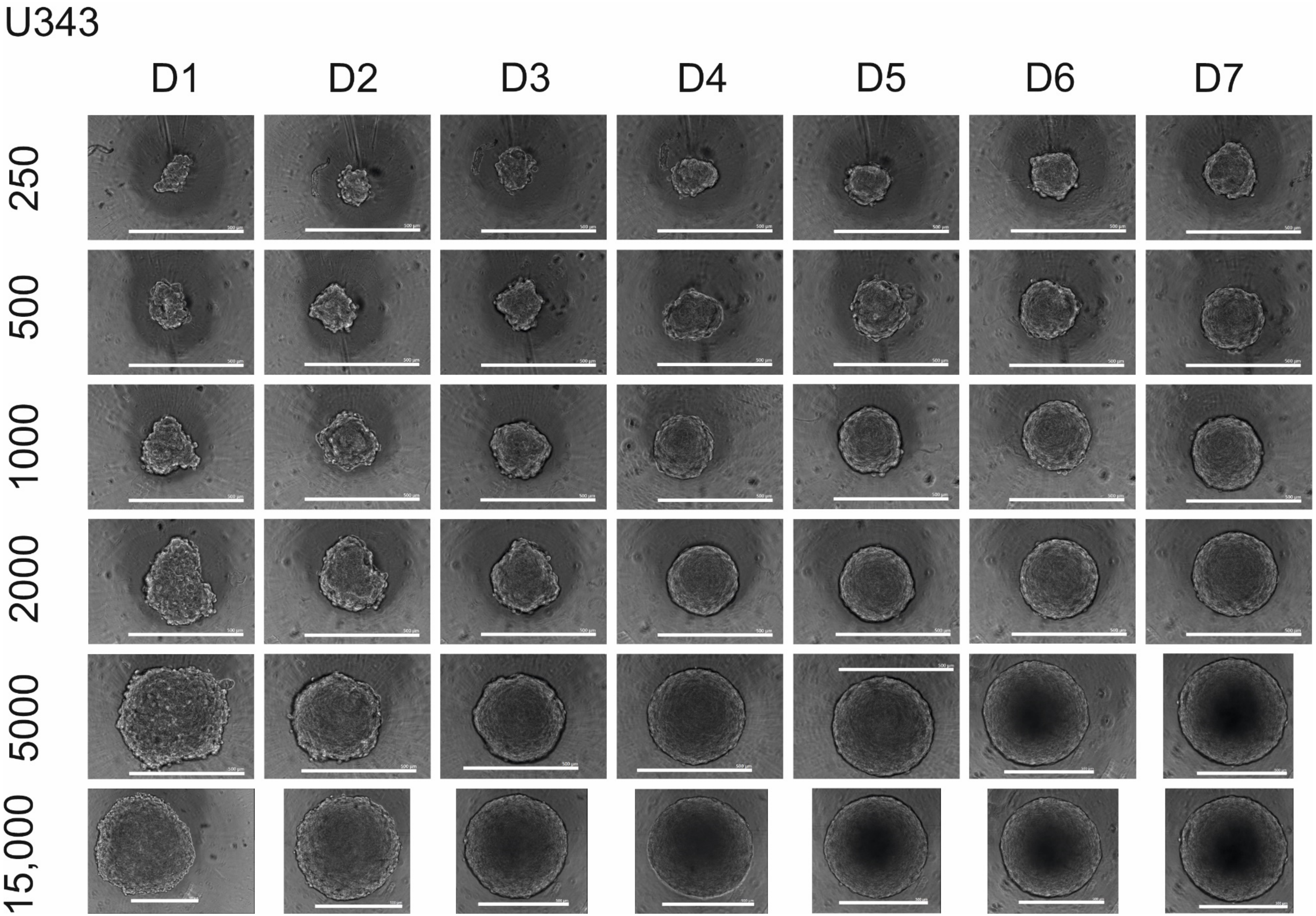
U343
D1
D2
D3
D4
D5
D6
D7
250
500
1000
2000
5000
15,000
