# Supplementary material for: Rapid and reproducible generation of glioblastoma spheroids for high-throughput drug screening
Source: Front Bioeng Biotechnol. 2024 Dec 18;12:1471012. doi: 10.3389/fbioe.2024.1471012 (PMC11688379; doi:10.3389/fbioe.2024.1471012)
Supplement: Supplementary file 5 [file DataSheet5.pdf]

Supplemental Table 3. Cell doubling times in standard medium.

The table presented shows cell culture doubling times in hours determined by life cell imaging in standard medium (DMEM with 4.5 g/L glucose, 10 % FCS, and 2 mM GlutaMAX) during exponential growth.

	Mean	SEM
1321N1	23.98	0.25
G55T2	26.23	0.32
LN229	24.12	0.29
LN405	46.15	0.34
MZ18	41.14	0.37
MZ54	27.79	0.29
T98G	29.08	0.18
U87	39.95	0.25
U251	30.92	1.08
U343	28.77	0.29
